# Protocol for a meta-narrative review on research paradigms addressing the urban built environment and human health

**DOI:** 10.1186/s13643-021-01848-6

**Published:** 2021-12-11

**Authors:** Jinhee Kim, Ben Harris-Roxas, Evelyne de Leeuw, David Lilley, Alana Crimeen, Peter Sainsbury

**Affiliations:** 1 Centre for Health Equity Training, Research and Evaluation (CHETRE), Part of the UNSW Australia Research Centre for Primary Health Care & Equity, A Unit of Population Health, South Western Sydney Local Health District, NSW Health, A member of the Ingham Institute, Sydney, Australia; 2 School of Population Health, University of New South Wales, Sydney, Australia; 3 Healthy Urban Environments (HUE) Collaboratory, Maridulu Budyari Gumal Sydney Partnership for Health, Education, Research and Enterprise SPHERE, Sydney, Australia; 4 City Futures Research Centre, UNSW Arts, Design & Architecture, Sydney, Australia; 5 UNSW Arts, Design & Architecture, Sydney, Australia; 6 School of Medicine, University of Notre Dame Australia, Sydney, Australia

**Keywords:** Paradigms, Urban health, Healthy cities, Healthy urban planning, Health social movements, Medical-industrial complex, Meta-narrative review

## Abstract

**Background:**

Urban health is a field of research and practice that has attracted the interest of various disciplines. While it is encouraged for diverse disciplines to contribute to a multidisciplinary field of study such as urban health, this often results in tensions, conflicts or competition between the different traditions that stem from different epistemological backgrounds.

This meta-narrative review aims to identify and describe the multiple paradigms and articulate the underlying epistemological, ontological, methodological, and aetiological differences in their approaches. Articulating the paradigms not only contributes to the advancement of research, but also provides a framework for understanding the different policy beliefs and ideas policy actors hold and apply in the policy process.

**Methods:**

We apply the meta-narrative method to systematic literature review which includes the following six iterative phases. The planning phase includes the finalisation of the review protocol and assembly of review team. The search phase includes a comprehensive literature search in key databases and a double-sided systematic snowballing method. We will search multidisciplinary databases including Web of Science, Scopus and ProQuest, and topic-specific databases including Urban Studies Abstracts (EBSCO), MEDLINE, and EMBASE from their inception onwards. Bibliometric analyses of this literature will be used to triangulate the mapping of the paradigms. The mapping phase includes identifying the dominant paradigms and landmark publications through agreement with the review team. In the appraisal phase, the literature will be assessed by their respective quality standards, followed by data extraction to identify the individual narratives in the conceptual, theoretical, methodological, and instrumental dimensions of each paradigm. The synthesis phase will review the data to compare and contrast and identify the overarching meta-narratives. The recommendation phase will include dissemination of the findings from the review.

**Discussion:**

The meta-narrative review will reveal the how the different paradigms conceptualise, frame and prioritise urban health issues, their preferred methodologies to study the phenomenon, and the nature of the solutions to improve human health. This review will assist researchers and practitioners in understanding and interpreting evidence produced by other traditions that study urban health. Through this, urban health researchers and practitioners will be able to seek coherence in understanding, explaining, and exploring the urban health phenomenon.

**Systematic review registration:**

Open Science Framework (https://osf/io/tn8vk)

**Supplementary Information:**

The online version contains supplementary material available at 10.1186/s13643-021-01848-6.

## Background

The research and practice of urban health involves contributions from multiple disciplines, sectors, and trades that represent different aspects of urban health phenomena. Each discipline, sector or trade has a role in the complex interplay between the urban built environment and its relationship to health and health equity impacts. While it is encouraged for actors from diverse backgrounds to contribute to a multidisciplinary field of study such as urban health, collaboration efforts sometimes result in tensions, conflicts, and competition. This is because disciplines and sectors have historically evolved in silos and branched out as specialisations that have developed different standards of ‘normal science’, or practices or empirical approaches that members of a certain discipline or sector take for granted [1]. The different standards are strongly rooted in the epistemological, ontological, methodological, and aetiological definitions of urban health of the diverse disciplines that often act as barriers to meaningful interdisciplinary or intersectoral collaboration [2–4].

These positions are defined as *paradigms*, or particular frameworks that researchers apply to understand the complexity of the real world. In a given paradigm, a distinct set of concepts and practices provides a common framework for addressing problems and solutions [1]. The set of concepts of a paradigm defines what is regarded as important issues that require attention in urban health and which study designs and methods are the best ways to produce the required knowledge. Therefore, a group of researchers and practitioners that belong to a paradigm share a set of rules and standards that are self-evident but are incommensurable with other paradigms. That is, empirical findings that were produced using one set of concepts, theories, methods, and instruments may not only be inapplicable to issues seen important in other paradigms but unacceptable to the followers of a different paradigmatic lens.

In our preliminary review of the literature, we identify four prominent paradigms in the study and policies that address the impact of the urban built environment on human health. The approaches of the ‘*medical-industrial city*’ paradigm focus on the development of healthcare facilities as a key urban planning project in the city or the application of technology to the urban infrastructure to monitor or change disease, risk factors, and behaviours of individuals. The *‘urban health science’* paradigm applies epidemiological and complex systems analyses to urban health issues. Here, conclusive empirical data and analyses that confirm the causal relationships between the urban built environment and human health outcomes is prioritised and is used as evidence to develop effective interventions and policies. The ‘*healthy built environment*’ paradigm originates from the urban planning discipline and advocates for the integration of health in the practice of spatial planning of cities. The ‘*health social movement*’ paradigm seeks to integrate health considerations into all aspects of urban governance, with an emphasis on operationalising values such as health equity and empowerment. This typology has been developed from an earlier version in which we had identified the latter three paradigms [5].

Other studies have similarly confirmed the co-existence of multiple approaches in this field. From the articles retrieved via a search in PubMed/MEDLINE of the MeSH term “urban health”, Jia et al. [6] identified four distinct categories of urban health research as physical environment, health impacts, social environments and interventions. Forsyth [7] identified three conceptually distinct categories of healthy places approaches, i.e. basic healthy places (developing a physical and/or institutional structure supportive of health), population-based lenses (focus on population groups with health vulnerabilities and wide relevance), and technology-focused places (harnessing innovative technology to create a healthy economy and/or assist in health monitoring and promotion).

In contrast to the distinctions in subject matter made by the above authors, our emphasis is on identifying and describing the multiple paradigms and articulating the underlying epistemological, ontological, methodological, and aetiological differences in their approaches. This is particularly important for the field of urban health because maximising health gains cannot be effectively achieved by merely working within the common intersecting areas of specific disciplines and sectors. Urban health is a multidisciplinary field of research and practice that requires more attention and understanding in the non-overlapping areas. This meta-narrative review attempts to study how the topic of the urban built environment and human health has been differently conceptualised and researched by different traditions.

Articulating the paradigms not only contributes to the advancement of research, but also provides a framework for understanding the different policy beliefs and ideas policy actors hold and apply in the policy process. Ideas are organised into policy paradigms which sometimes have the power to induce changes to the institutional routines [8]. Moreover, understanding the key dimensions of the different urban health paradigms can help to prevent answering the research question correctly but forming a wrong interpretation or response [9].

For the purpose of this study, we limit the scope of the urban health phenomenon to approaches in research and practice that address the issues concerned with the impact of the urban built environment on human health in cities or urban areas. In this meta-narrative review, we aim to identify the different contemporary urban health paradigms and articulate the characteristics in their conceptualisation, theoretical framework, methodological approaches and instrumental solutions to urban health issues. The units of analysis are the different urban health paradigms and publications are the data source to examine their characteristics.

The primary research question of this review is: What are the dominant paradigms in research and practice on the issue of the urban physical environment and its impact on human health? Secondary research questions involve identifying the characteristics typical of each paradigm, such as (a) What are considered the important objects of study in each paradigm? (b) How are urban built environments and their impact on human health conceptualised? (c) Which methodological approaches are preferred? (d) What is the nature of the policy solutions?

## Methods/design

To understand the heterogeneity of research traditions that study urban health, we apply the meta-narrative approach to systematic literature review. A meta-narrative review is a type of systematic literature review that is designed for topic areas that are researched by diverse research traditions, with different conceptualisations and methodologies [10–13]. Through an explicit, rigorous, and transparent process, meta-narrative reviews identify, articulate, synthesise, and interpret a diverse body of literature in a topic area [11, 14, 15]. In this review, we will systematically collect and analyse the literature that addresses the issues concerning the impact of urban built environments on human health and seek to make sense of the complex and contested knowledge in this topic area.

The protocol for this review was developed in accordance with the RAMESES (Realist And MEta-narrative Evidence Syntheses: Evolving Standards) publication standards [15] and quality criteria suggested in the associated meta-narrative review training materials [14]. Because meta-narrative reviews are different from traditional systematic literature reviews that they are designed to reflect the heterogeneity of the research methodologies, the protocol is not fully compatible with the Preferred Reporting Items for Systematic Review and Meta-Analysis Protocol (PRISMA-P) guidelines. Nevertheless, we have populated the PRISMA-P checklist as it still provides critical value to the systematic review process (Additional file 1). This study protocol has been registered within the Open Science Framework (registration number: https://osf.io/tn8vk)

The six guiding principles of the meta-narrative review [15] are integrated into the review process as articulated below (Table 1).

**Table 1:** Meta-narrative review principles and applications

Principle	Definition	Application in this review
***Pragmatism***	The review should address what will be most useful to the intended audience	The objective of this review is to understand the main paradigms in urban health. In a transdisciplinary field of research and practice, articulating the non-overlapping characteristics of different paradigms is critical to attain coherence and collaboration across disciplines, sectors, and paradigms.
***Pluralism***	The topic should be illuminated from multiple angles and perspectives	We explore the current knowledge base in various disciplines, including public health, urban planning, local/city governance, and urban studies. A list of relevant disciplines and journals will be drafted to utilise for the hand selecting of literature to avoid any exclusion of the disciplines.
***Historicity***	The deepest understanding of a topic comes from studying its evolution over time	The genealogy and clusters of the literature will be analysed using bibliometric methods. Landmark documents will be recorded and traced to study the evolution of the paradigms.
***Contestation***	Conflicting data from different research traditions should be examined to generate higher-order insights	Differences between the conceptualisation of urban health, causal pathways, methodological approaches, and policy solutions will be highlighted. Details on the application of this principle will be explored further in the data extraction, analysis, and synthesis phases.
***Reflexivity***	Reviewers should continually reflect on the emerging findings	The protocol will be updated to reflect the changes to the process as findings emerge. Any changes made to the review that were initially planned will be described and justified in the final report.
***Peer review***	Emerging findings should be presented and discussed with an external audience	The emerging findings will be communicated with peers via individual consultations with experts and presentations at conferences and meetings. A website will be developed as a platform for the wider community. to engage in the process as well as for dissemination of the findings.

The methods section of this protocol is presented according to the six phases of a meta-narrative review recommended by the RAMESES publication guideline [15].

### Planning phase

A review team, consisting of the authors of this protocol, developed the research questions and drafted the protocol. Knowledge users, defined as the broader network of researchers and practitioners in the field of urban health, will be approached to serve as external expert panel members for the review and will be consulted if additional assistance is needed.

### Information sources and search strategy

#### Search strategy

The main objective of the search is to collect a comprehensive list of the literature on the topic area to capture the diversity of the urban health research traditions and paradigms. The publications will serve as the primary data source to analyse the meta-narratives of each paradigm. The balance between comprehensiveness and precision of the search is resolved by applying the concept of saturation as the criterion, a concept borrowed from qualitative research methods. Saturation is achieved when no new insights are generated by collecting additional data. Because this review is a ‘knowledge-building and theory-generating’ type of systematic review, there is no intrinsic value in continuing the search unless there is additional theoretical contribution [16]. This is in contrast to those reviews which study the aggregation or summation of concepts, where ‘the more the better’ approach is preferred. The attainment of saturation will be determined at the appraisal or synthesis phase when the review team observes a conceptual saturation of the findings from the identified literature, and decides that the addition of unidentified studies will only contribute to marginal changes to the findings [17–19].

The search will take three main strategies—(a) a double-sided snowballing search, (b) a search in electronic databases using search terms, and (c) an additional hand search. The double-sided snowballing search will include a forward search of all papers that cite the landmark works identified in the mapping phase, and a backward search that collects the literature included in the reference lists of these papers [20]. A search using keyword search terms will be conducted in relevant multidisciplinary scientific databases (e.g. Web of Science (Science and Social Sciences Citation Indices), Scopus, ProQuest) and topic-specific databases (e.g. Urban Studies Abstracts—EBSCO, Medline—Ovid, Embase_Ovid). The search terms will include those related to city (“city” OR “cities” OR “urban” OR “local” OR “municipal”) combined with concepts on the built environment (“built environment” OR “physical environment” OR “infrastructure” OR “planning” OR “design”) and search terms related to health and health equity. A draft search strategy for Web of Science that has been reviewed by an information specialist is available in Additional file 2. However, because we aim to perform a precise search that captures the diversity of the approaches on urban health rather than a sensitive or comprehensive one, the search terms and inclusion criteria need to remain flexible and porous. An additional hand search of key journals and publications by key organisations will be conducted to maximise comprehensiveness.

#### Selection process

The publications identified from these three strategies will be compiled in EndNote and exported to Covidence to be screened for inclusion in the review. Two reviewers will screen the title and abstract of each publication to decide inclusion in the review. Any disagreement will be resolved by consensus. Details of the inclusion criteria will be added and refined as the reviewers proceed with the screening. It is also expected that the search strategy will be iteratively revised by the paradigms identified in the mapping phase.

#### Eligibility criteria

Publications to be included in the review will be limited by language (English) and publication types (journal articles, reviews, books, book chapters, editorial and opinion pieces, and reports). All study designs, including empirical (e.g. observational studies, quantitative studies, mixed methods) and non-empirical studies (e.g. reviews, conceptual papers) in all publication years will be considered for inclusion. The topic of the paper must explicitly focus on urban human-constructed physical environments and human health at the city or urban scale and must address one or more of the conceptual, theoretical, methodological or instrumental dimensions on this topic. To be eligible for inclusion, a publication must address all three concepts—urban, built environment, and health. The topic of the publication must address the interface of the built environment and human health at the “urban” scale that includes cities in terms of size, population, density, level of government, administration, and urban morphological features. “Built environment” includes the physical human-made morphological features of urban areas such as infrastructure, buildings, streets, and the systems processes that shape the decision-making such as urban planning policies and processes. The concept of “human health” not only includes the health behaviours and health outcomes of individuals and communities, but broader concepts such as health equity, liveability, resilience, and sustainability at the urban scale.

### Mapping phase

#### Mapping urban health paradigms and defining parameters

As mentioned earlier, we start with four paradigms on urban health—the medical-industrial city, urban health science, healthy built environments and health social movement—as the initial paradigms for this review (Table 2). These four paradigms explicitly address the relationship between the urban built environment and human health, and each has a set of conceptual, theoretical, methodological, and instrumental dimensions. Findings from the search and discussions with the wider expert community may introduce additional paradigms to the review. For example, there may be additional paradigms in the fields of environmental health, spatial justice, or civil engineering which were not covered in our initial mapping. Alternatively, the initial paradigms may merge or subdivide as more information is added from the review process.

**Table 2:** Four initial urban health paradigms

	Medical-industrial city	Urban health science	Healthy built environments	Health social movements
Main concept	The healthcare industry is a main driver for urban growth and development.	Production of evidence that reveal and confirm causal relationships between the built environment and health.	Influencing the planning system to integrate health to urban planning decisions.	Empowering the community and value-based decision-making for healthy cities.
Related disciplines and fields	Business, urban planning, development, economics	Public health, epidemiology, population health, implementation science	Urban planning, urban design, land-use planning	Health promotion, community development, social justice, sociology

In the mapping phase, we will develop a set of parameters for each paradigm. For example, we will define the characteristics of each paradigm based on their definitions of urban health, the theoretical frameworks that explain the relationship between urban physical environments and health, the methodologies to research (e.g. what is counted as knowledge, evidence) and the solutions such as the policies, strategies, and practices to improve urban health. In particular, we will include the concepts of health equity and the consideration of power in decision-making in reviewing the parameters. These parameters will be used as a guide to search for data on the dimensions of the multiple paradigms in each publication. We will apply the set of parameters to assign each piece of an included publication to its corresponding paradigm(s). To ensure high inter-coder reliability, the review team will first code a small sample of the publications and any conflicts or disagreement will be examined and discussed. Any disagreement will be resolved by consensus within the review team.

#### Bibliometric analysis to visualise network and clusters

A supplementary bibliometric analysis using the dataset of the final set of articles will be conducted to map the genealogy of citations and conduct a social network analysis of the authors [21]. Co-authorship and document co-citation network analysis will visualise clusters of researchers and relationships between publications that provide information on research groups, themes, and the overall scientific landscape. Co-authorship networks are based on the frequency of authors who co-author a publication while a document co-citation relationship occurs when two publications are cited by a third publication. Clusters of a close co-citation relationship can be interpreted as belonging to the same ‘research front’ [22]. These visualisation data will provide information to triangulate the different paradigms and research traditions.

#### Identifying landmark works

The mapping phase includes identifying landmark works that formed the foundation for the paradigms and are recognised by scholars in the field as highly influential in shaping subsequent research and practice [11]. They can be conceptual papers or reports, or empirical studies that formed a model for future work in the paradigm. We will triangulate this with the citation metrics data and the findings from the bibliometric network analysis.

The following inclusion criteria will be applied to identify the landmark sources [11]

1. Is the paper part of a recognised paradigm, that is, does it draw critically and comprehensively upon an existing body of scientific knowledge and attempt to further that body of knowledge?
2. Does the paper make an original and scholarly contribution to research and practice into the topic area?
3. Has the paper subsequently been cited as a landmark contribution (conceptual, theoretical, methodological or instrumental) by competent research and practice in that tradition?
4. Is the paper an exemplar of a recognised paradigm and its parameters?

The review team will independently score and nominate landmark sources according to the above criteria. Discussions will be held with external experts to attain consensus.

### Appraisal phase

In the appraisal phase, we will extract data from selected publications by coding the conceptual, theoretical, methodological and instrumental concepts. The main outputs from this phase include a codebook with the descriptions of the codes, an NVivo project with coded data of the included literature, development of the quality assessment criteria for each paradigm and the quality assessment of the literature.

#### Data extraction

First, a code system (or a data extraction template) will be developed based on the parameters of the paradigms developed in the mapping phase. The code system and data extraction template will include the following:

- Bibliometric meta data (e.g. author, publication year, title, type of publication)
- Research questions and how they were framed, and conceptual and theoretical issues;
- Preferred methodologies, study designs, and quality criteria;
- Key actors (e.g. leading scientists or commentators) and events (e.g. conferences) in the unfolding of the tradition;
- Landmark empirical or theoretical studies;
- Significant findings and how they shaped subsequent work;
- Key debates and areas of dispute within the tradition, including links with or breaches from other traditions;
- Characteristics in cross-disciplinary approaches (interdisciplinary, multidisciplinary or transdisciplinary).

Details of the coding system will be determined by the review team. Whether data extraction will be based on the abstract or the full-text will be determined at a later time, after the review team learns more about the body of literature. We will use the NVivo qualitative data analysis software to efficiently organise the data. Using NVivo software for qualitative coding will also allow us to refer back to the original data and transparently track the collaborative process. The reviewers will independently extract data and the coded data will be examined to ensure inter-coder reliability. All data will be stored in the approved research data storage system provided by the lead author’s institution and handled in accordance with the institution’s data management standards and guidelines.

#### Quality and risk assessment

It is an inherent property of paradigms that each will endorse a different set of standards for assessing the quality and risk of bias of studies. Criteria to assess the quality and risk of bias will be taken from the paradigms included in the review, particularly from the landmark papers that have been accepted by the paradigm as authoritative. The publications, now classified to one or more paradigm, will be assessed against the corresponding quality criteria. The included publications will be critically appraised for methodological quality using the Mixed Methods Appraisal Tool (MMAT) [23] for peer-reviewed journal articles and the AACODS (Authority, Accuracy, Coverage, Objectivity, Date, Significance) Checklist [24] for grey literature. To ensure consistency, all reviewers will discuss the applicability of MMAT and AACODS tools and assess a sample of full-text publications. Publications not included in the sample will be independently assessed by two reviewers. If all reviewers agree, publications that have been assessed as low quality may be excluded from the analysis.

### Synthesis phase

#### Building the meta-narratives of each paradigm

Synthesis involves comparing and contrasting the meta-narratives among the different paradigms to identify and compare how they have conceptualised the topic, how they have theorised it, and the methodological approaches and study designs used. To achieve this, each publication will be coded for the urban health sub-topic that the study addresses, concepts and theories the study is grounded in, methodologies, and key findings. The coded data will be checked by a second reviewer to attain consistency. The reviewers will iteratively search for patterns in the analysis, based on the four urban health paradigms that were identified in the preliminary literature review. The data will be analysed and categorised into thematic groups to present the individual accounts of the urban health issue, terminologies, definition of key concepts, theories on the causal pathways, preferred methods and key empirical findings of each group. This categorisation will be compared with the bibliometric network patterns. The findings will be discussed and agreed by all reviewers.

#### Comparing meta-narratives across paradigms

The purpose of comparing the meta-narratives across paradigms is not intended to develop a single theory, but rather, to highlight the diversity and articulate the commonalities and differences. Therefore, synthesis across paradigms may occur at a high level of abstraction and may involve one or more of the following [15]:

- Paradigm bridging (seeking commonalities in conceptual and theoretical assumptions)
- Paradigm bracketing (highlighting differences in these assumptions)
- Interplay (exploring tensions)
- Meta-theorizing (exploring patterns that span conflicting understandings)

Through a series of workshops, reviewers will iteratively compare the meta-narrative developed for each paradigm against the conceptual, theoretical, methodological and instrumental dimensions that define a paradigm. The key questions we will ask in this phase include (1) What is the range of questions the paradigms address across the four dimensions? (2) What are the commonalities and conflicts of research findings across the paradigms and how can the discrepancies be explained? (3) What are the overall key findings and implications? (4) What are the main gaps and where should future research be directed?

### Recommendation phase

The final phase of the review includes drafting the final report with key messages and recommendations for practice, policy, and further research. The final report will be developed through reflection and discussions with the review team and feedback from the wider urban health epistemological community. Reporting will be in compliance with the RAMESES publication standards [15]. Any changes made to the protocol will be documented in the final review report.

## Discussion

To our knowledge, there has not been a comprehensive search of the literature that identifies the different approaches to urban health issues and their solutions. By systematically reviewing the literature, we will be able to identify the different paradigms within which researchers and policy actors address urban health issues and develop a comprehensive map of the field. Paradigms are not only relevant in research and science, but also have a fundamental role in forming the policy ideas and beliefs of the actors involved in the policy process [8, 25–28]. The findings from this review will contribute to highlighting conflicting evidence between the paradigms and finding gaps in the approaches. Detailed articulation of the paradigms will facilitate communication and knowledge transfer between previously incommensurable paradigms.

Since this meta-narrative review is iterative by nature, we expect the protocol to continually evolve and reflect the emerging findings and feedback from the wider epistemic community. Any amendments made to this protocol when conducting the study will be outlined and justified in the final report of this meta-narrative review. A dissemination strategy will be developed to further communicate with the broader knowledge user community. Components of the review (e.g. the protocol, methodology, meta-narrative review findings) will be reported through publications in peer-reviewed academic journal publications, conference presentations, interactions with potential knowledge users. Presenting the findings of the review over the various stages will provide a form of triangulation to ensure the validity of the review and address some of the issues occurring from meta-biases such as publication bias or selective reporting. The team will further identify a broad range of potential knowledge users and stakeholders and develop innovative strategies to effectively communicate with them.

In the development of this protocol, we adhered to the RAMESES publication guideline and its quality standards [15]. However, in some cases, we found that the meta-narrative approach that we apply in our review is not completely compatible with the required reporting templates for systematic reviews such as PROSPERO, the International Prospective Register of Systematic Reviews, or the Preferred Reporting Items for Systematic Review and Meta-Analysis Protocol (PRISMA-P). For example, because the units of analysis of a meta-narrative review are paradigms, it is not appropriate to identify the PICO elements—Participants, Intervention, Comparison, Outcomes—which are elements typical of systematic reviews that study the effectiveness of interventions often in clinical settings. Also, because the execution of meta-narrative reviews is iterative by nature, that is, findings from a subsequent phase will often provide information about or for a previous phase, these processes are often challenging to describe in the templates that were designed for a more linear process. Similarly, as we plan to take a reflective and interpretive approach by presenting our preliminary findings to other colleagues within the broader urban health research community for their feedback throughout the different stages of the review, these iterative and interpretive characteristics were not effectively captured in the PROSPERO or PRISMA-P reporting guidelines. Lastly, because meta-narrative reviews include studies across different paradigms and study designs, the criteria applied to assess the studies must inevitably be selected according to the standards of each paradigm and research tradition.

In summary, this meta-narrative review will reveal how the different paradigms conceptualise, frame and prioritise urban health issues, their preferred methodologies to study the phenomenon, and the nature of the solutions to improve human health. The findings from the review will assist researchers and practitioners in understanding and interpreting evidence produced by paradigms other than their own that study urban health. Through this, urban health researchers and practitioners will be able to seek coherence in understanding, explaining, and exploring the urban health phenomenon.

## Supplementary Information

**Additional file 1.** PRISMA-P checklist.

**Additional file 2.** Search strategy.

## Data Availability

Not applicable

## Abbreviations

AACODS: Authority, Accuracy, Coverage, Objectivity, Date, Significance
MMAT: Mixed Methods Appraisal Tool
PRISMA-P: Preferred Reporting Items for Systematic Review and Meta-Analysis Protocol
RAMESES: Realist And MEta-narrative Evidence Syntheses: Evolving Standards
