# Supplementary material for: Protocol for a meta-narrative review on research paradigms addressing the urban built environment and human health
Source: Syst Rev. 2021 Dec 11;10:311. doi: 10.1186/s13643-021-01848-6 (PMC8665591; doi:10.1186/s13643-021-01848-6)
Supplement: Supplementary file 2 — Additional file 2. Search strategy. [file 13643_2021_1848_MOESM2_ESM.docx]

The search terms and inclusion criteria need to remain flexible, porous, and inclusive of the diverse research paradigms. Therefore, other than the basic criteria that set the boundaries for the review, such as publication type (journal articles, books and reports), year (all years), language (English), the search strategy and criteria were revised as new information emerged from the retrieved publications.

We will apply a text word search using synonyms for three major concepts (urban, built environment and health). We plan to restrict the search fields to title, abstract and author keywords and used Boolean operators to link synonyms and exclude non-relevant topics. New search terms will be added and refined in an iterative process of reviewing the list of searched documents and adding variations of synonyms and excluding non-relevant search terms. A draft search term is shown in the table below.

Table 1 Search terms

| Concept | Search terms |
| --- | --- |
| URBAN | (city OR cities OR local government OR municipal OR municipality OR megacity OR megacities OR urban OR urbanity OR urbanicity OR metropolitan OR metropolis OR urbanisation* OR urbanization*) NOT rural |
| BUILT ENVIRONMENT | (“built NEAR/3 environment*” OR “urban NEAR/3 plan*” OR “infrastructure*” OR "physical environment*" OR “morphology” OR “morphologies” OR “urban NEAR/3 form” OR architecture OR design OR space OR place OR plan OR plans OR policy OR policies) NOT (“family planning” OR “health planning”) |
| HEALTH | (health OR healthy OR unhealthy OR "ill-health" OR "ill health" OR wellbeing OR “well being” OR well-being OR liveable OR liveability OR resilient OR resilience OR sustainable OR sustainability) NOT (“healthcare” OR “health care” OR “health-care” OR medical OR medicine OR “health service*” OR “health center*” OR “health centre*” OR sexual) |
